# A native promoter and inclusion of an intron is necessary for efficient expression of GFP or mRFP in *Armillaria mellea*

**DOI:** 10.1038/srep29226

**Published:** 2016-07-07

**Authors:** Kathryn L. Ford, Kendra Baumgartner, Béatrice Henricot, Andy M. Bailey, Gary D. Foster

**Affiliations:** 1 School of Biological Sciences, Life Sciences Building, University of Bristol, 24 Tyndall Avenue, Bristol, BS8 1TQ, United Kingdom; 2 United States Department of Agriculture-Agricultural Research Service, 363 Hutchison Hall, University of California, One Shields Avenue, Davis, CA 95616, United States; 3 The Royal Horticultural Society, Wisley, Woking, Surrey, GU23 6QB, United Kingdom

## Abstract

*Armillaria mellea* is a significant pathogen that causes Armillaria root disease on numerous hosts in forests, gardens and agricultural environments worldwide. Using a yeast-adapted pCAMBIA0380 *Agrobacterium* vector, we have constructed a series of vectors for transformation of *A. mellea*, assembled using yeast-based recombination methods. These have been designed to allow easy exchange of promoters and inclusion of introns. The vectors were first tested by transformation into basidiomycete *Clitopilus passeckerianus* to ascertain vector functionality then used to transform *A. mellea*. We show that heterologous promoters from the basidiomycetes *Agaricus bisporus* and *Phanerochaete chrysosporium* that were used successfully to control the hygromycin resistance cassette were not able to support expression of mRFP or GFP in *A. mellea*. The endogenous *A. mellea gpd* promoter delivered efficient expression, and we show that inclusion of an intron was also required for transgene expression. GFP and mRFP expression was stable in mycelia and fluorescence was visible in transgenic fruiting bodies and GFP was detectable *in planta*. Use of these vectors has been successful in giving expression of the fluorescent proteins GFP and mRFP in *A. mellea*, providing an additional molecular tool for this pathogen.

*Armillaria mellea* (honey fungus) is an important and virulent pathogen that has a global distribution^1^. With a broad host range of >500 species^2^, it causes significant damage in many horticultural, agricultural and forestry environments^3^,^4^,^5^,^6^. Armillaria root disease has been studied extensively for decades^7^,^8^,^9^,^10^,^11^, but it is only recently that a variety of molecular and genetic approaches have been developed for *Armillaria*. In particular, the genomes of *A. mellea*, *A. gallica* and *A. ostoyae* have been sequenced^12^,^13^, transcriptomic and proteomic data is available^13^,^14^, an *Agrobacterium tumefaciens*-mediated transformation system has been developed for *A. mellea*^15^ and conditions for fruiting have been established to provide the basidiospores needed for this transformation method^16^.

An additional molecular tool that has proven to be useful in other fungi, but is currently unavailable for *Armillaria*, is the expression of reporter genes. Reporter genes such as luciferase (LUC), (β-glucuronidase (GUS), green fluorescent protein (GFP) and red fluorescent protein (DsRed) and their derivatives have been used to analyse pathogen-host interactions *in planta*^17^,^18^,^19^, facilitate early detection of infection in pathogenicity assays^20^, permit promoter:reporter gene fusions to study gene expression patterns and localisation^21^,^22^ and to assess requirements for heterologous gene expression^23^,^24^,^25^,^26^. Enhanced green fluorescent protein (eGFP), adapted from GFP of the jellyfish *Aequorea victoria*, is one of the most frequently used reporter genes for expression in Agaricomycetes^21^,^23^,^27^,^28^, owing to its non-destructive visualisation, sensitivity, stability and activity independent of cofactors or additional substrates^29^.

Earlier transformation work with *A. mellea*^15^ used a vector previously developed for *Agaricus bisporus*, pBGgHg^30^, containing intronless hygromycin resistance (*hph*) and GFP cassettes driven by the *A. bisporus* glyceraldehyde-3-phosphate dehydrogenase (*gpdII*) promoter with the *Cauliflower mosaic virus* (CaMV) 35S terminator. Although GFP expression was not the objective of the work, the authors noted that no fluorescence was observed^15^. The knowledge of expression requirements for basidiomycetes is limited, but studies with other Agaricomycetes have attributed inefficient transgene expression to poor codon optimisation^26^,^31^, ineffective promoters^23^,^26^,^32^,^33^ and the absence of an intron^23^,^25^,^28^,^34^. Determining the factors important for efficient transgene expression in basidiomycetes is important, not only for obtaining expression of reporter proteins, but also for permitting heterologous expression of other genes of interest, for example, to enable use of targeted gene-editing technologies such as the CRISPR/Cas9 system^35^,^36^ that is functional in yeasts^37^,^38^ and filamentous ascomycetes^39^,^40^,^41^,^42^ but has only been reported in two basidiomycetes to date^43^,^44^.

In this work, we construct a series of vectors designed to obtain GFP and mRFP expression in *A. mellea* and use the transformation-amenable antibiotic-producer *Clitopilus passeckerianus* to evaluate vector functionality. This work provides an additional molecular tool for *A. mellea* and assesses prerequisites for efficient heterologous expression in this important Agaricomycete.

## Methods

### Strains and culture conditions

*Armillaria mellea* (ELDO17) and *Clitopilus passeckerianus* (DSMZ1602) were maintained on potato dextrose agar (PDA) at 25 °C in the dark. *Saccharomyces cerevisiae* (Y10000) was used for plasmid construction by homologous recombination and was maintained on yeast peptone dextrose agar (YPDA) at 28 °C. *Escherichia coli* (DH5α) was used for subcloning plasmids. *Agrobacterium tumefaciens* strains AGL-1^45^ and LBA1126^46^ were used in *A. tumefaciens*-mediated transformation of fungi and were maintained on LBA at 37 °C and 28 °C, respectively.

### Vector construction and *Agrobacterium tumefaciens* transformation

**Figure 1:**
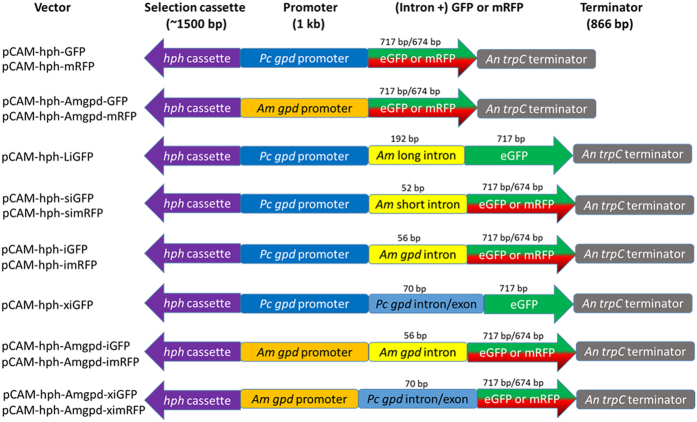
Schematic of the hph and eGFP/mRFP cassettes made in this study. The *hph* cassette was amplified from pBGgHg and has the *Agaricus bisporus gpdII* promoter driving *hph* with the CaMV 35S terminator^30^. *Armillaria mellea* (*Am*) long and short introns are introns 11 and 7, respectively, from putative efflux transporter genes (NCBI reference EF547153 and EF547152, respectively)^48^. The *Phanerochaete chrysosporium gpd* (*Pc gpd*) promoter, *P. chrysosporium* intron/exon, eGFP and the *Aspergillus nidulans trpC* terminator (*An trpC*) were amplified from pGR4-GFP or pGR4-4iGM3^23^. mRFP was amplified from pYES-hph-RFP004^24^. The sequence of the *A. mellea gpd* promoter (*Am gpd*) and intron was based on the gene model with protein ID 13125^13^.

Vectors were designed in Clone Manager (Sci-Ed Software) and were constructed by homologous recombination in yeast, based on the protocol in Gietz & Woods^47^ using the vector pCAM-hph-series previously described in Ford *et al*.^16^. Construction details for the vectors are in Supplementary Tables S1 and S2. pCAM-hph-series is based on a yeast-adapted pCAMBIA0380 vector and contains the hygromycin resistance cassette (*hph*) from pBGgHg^30^, where *hph* is driven by the *A. bisporus gpdII* promoter and is flanked by the CaMV 35S terminator. Regulatory sequences for GFP and mRFP in constructed vectors are either the *Phanerochaete chrysosporium* or *A. mellea gpd* promoter, both with the *Aspergillus nidulans trpC* terminator (Fig. 1). The *A. mellea gpd* promoter sequence was obtained from the sequenced *A. mellea* genome^13^, available online at genome.jgi.doe.gov/Armme1_1/Armme1_1.home.html (protein ID 13125), defined as 1 kb upstream from the start codon and amplified from *A. mellea* ELDO17. *Armillaria mellea* intron sequences, where included in vectors, were obtained from NCBI (EF547152 and EF547153; introns 7 and 11, respectively)^48^ and the *A. mellea gpd* (genome.jgi.doe.gov/Armme1_1/Armme1_1.home. html) (protein ID 13125; intron 1). Introns were either amplified from genomic DNA of *A. mellea* isolate ELDO17 or purchased as two complementary oligonucleotides using the sequences available on NCBI or the JGI database. Plasmids pGR4-4iGM3 and pGR4-GFP^23^ are based on pGreen and contain the truncated *hph* gene (where the first two lysine codons are deleted^25^) driven by the *A. bisporus gpdII* promoter with the *A. nidulans trpC* terminator and eGFP controlled by the *P. chrysosporium gpd* promoter and *A. bisporus gpdII* promoter, respectively, with a 5′ intron/exon region from *P. chrysosporium gpd*. eGFP is flanked by the *P. chrysosporium mnp* 3′ UTR in both pGR4-4iGM3 and pGR4-GFP.

The Zymoprep Yeast Plasmid Miniprep II (Zymo Research) kit was used to extract plasmids from *S. cerevisiae*, which were subsequently rescued into *E. coli* and verified by PCR using LB/RB primers (Supplementary Table S3), restriction digestion and sequence analysis. Plasmids were transformed into competent *A. tumefaciens* by electroporation. *Agrobacterium tumefaciens*-mediated transformation of *A. mellea* was performed as described by Baumgartner *et al*.^15^ using basidiospores from *in vitro*-produced fruiting bodies of *A. mellea* isolate ELDO17^16^ and following the protocol of Kilaru *et al*.^25^ for transformation of *C. passeckerianus* mycelium. Putative transformants were verified by serial subculture to PDA supplemented with 200 μg/ml timentin and 30 μg/ml or 50 μg/ml of hygromycin for *A. mellea* and *C. passeckerianus*, respectively. Transgene presence was confirmed by PCR analysis with *hph*, GFP and mRFP primers (Supplementary Table S3).

### Microscopy for visualisation of fluorescent mycelia

Visualisation of fluorescence was attempted in all hygromycin-resistant *A. mellea* and *C. passeckerianus* colonies grown in potato dextrose broth (PDB) or on PDA for 1–2 weeks using a Leica DM LB microscope fitted with an excitation filter of 450–490 nm, dichroic filter of 510 nm and an emission filter of 515 nm for GFP and using an excitation filter of 545 nm with an emission filter of 610 nm for mRFP. Images were captured with a Nikon Coolpix 900 camera.

### Plant propagation and inoculation

To assess GFP and mRFP expression *in planta*, walnut grown in tissue culture^49^ was inoculated with three transformants: one GFP-expressing transformant (ELDO17-Amgpd-xiGFP2) generated with vector pCAM-hph-Amgpd-xiGFP, one mRFP-expressing transformant (ELDO17-Amgpd-ximRFP1) generated with vector pCAM-hph-Amgpd-ximRFP and one non-expressing transformant (ELDO17-siGFP1) generated with vector pCAM-hph-siGFP. Plantlets of walnut rootstock EA16 [*Juglans microcarpa* DJUG31.09 × *J. regia* (open pollinated)] were derived from micropropagated shoot cultures, multiplied and rooted *in vitro* on agar-based growth medium (Driver Kuniyuki Walnut medium) in Magenta Corp GA7 boxes (75 × 75× 100 mm)^50^. There were four plantlets per Magenta box, rooted in a 1.5 cm-thick layer of medium for two weeks, until at least three, 1-cm-long roots per plant formed. Inoculum was prepared by growing each isolate in PDB for 1 week (25 °C, 100 rpm, homogenising the mycelium for 30 s, and then transferring with a sterile 1 ml glass pipette 100 μl homogenate (i.e. mycelial fragments) per plant onto the surface of the medium. 100 μl sterile PDB were used to mock-inoculate control plants. There were three replicate Magenta boxes per transformant. Inoculated plants were incubated at 25 to 27 °C with a 16 h photoperiod provided by fluorescent light (F72T12/CW/VHO, Philips Lighting Company, Sommerset, NJ).

After 6 weeks, at which point symptoms of Armillaria root disease were expressed among inoculated plants, infection was confirmed by recovery of the pathogen in culture. Roots from all Magenta boxes (both inoculated and non-inoculated) were carefully separated from the medium and two to four root tips per plant were plated on water agar. After 10 d incubation at 25 °C, cultures were inspected for presence of *A. mellea* colonies with the following characteristics: ~2 cm colony diameter, regular colony margin, sparse white aerial hyphae, clampless hyphae embedded in the agar, absence of spores/spore-bearing structures and the possible presence of immature rhizomorphs.

### Confocal microscopy for visualisation of fluorescence *in planta*

Six weeks post inoculation, root tips <2 mm in diameter (i.e. within the scanning limit of the confocal microscope) were sliced in cross-section by hand with a double-edged razor blade (~0.3 to 0.5-mm thick sections) and placed in phosphate buffered saline (PBS). Root sections were separated into three subsets for: 1) treatment with Alexa Fluor 488 - wheat germ agglutinin conjugate (WGA-AF 488; LifeTechnologies, USA), which binds non-selectively to fungal hyphae; 2) treatment with GFP Tag Antibody (Rabbit Polyclonal), Alexa Fluor 488 conjugate (antiGFP-AF 488; LifeTechnologies, USA), which binds to hyphae of GFP-expressing transformants; and 3) non-treated. Treated root sections were soaked for 1 h in 20 μg/ml WGA-AF 488 or antiGFP-AF 488, washed twice in 0.1 M phosphate buffer, pH 6.8 (30 min per wash), then mounted on microscope slides and covered with a glass coverslip. Non-treated root sections were soaked for 1 h, then washed twice, in 0.1 M phosphate buffer. Root sections were imaged with a Leica TSP SP2 confocal microscope with excitation at 488 nm and detection at 500–520 nm for GFP, WGA-AF 488, and antiGFP-AF 488, or with excitation at 561 nm and detection at 570–610 nm for mRFP. Under both excitation wavelengths, detection of root cells was at 664–996 nm. All root sections were scanned at 100×, a sequential series of images was collected from upper to lower root surfaces, and images were combined as a maximum projection, using FIJI (v2.0.0-rc-46, National Institutes of Health, Bethesda, MD USA).

### *In vitro* fruiting bodies

Fruiting was induced in one GFP-expressing *A. mellea* transformant (ELDO17-Amgpd-xiGFP2) and one mRFP-expressing transformant (ELDO17-Amgpd-ximRFP1) as described in Ford *et al*.^16^. Briefly, RST medium (30 g rice, 15 g sawdust, 150 ml deionised water with a 1 cm top layer of homogenised tomato) was inoculated with mycelial agar plugs from a four-week *Armillaria* culture and colonised at room temperature in the dark for four weeks. There were three replicate fruiting pots per transformant. Following colonisation, cultures were incubated at 23 °C, 125 μmol m^−2^ s^−1^ light, 70% RH, 16 h photoperiod for six weeks, followed by a reduction in light and temperature to 15 °C, 5 μmol m^−2^ s^−1^ light, 70% RH, 10 h photoperiod to induce primordia and allow development of fruiting bodies.

## Results

### Vector construction and analysis

Fourteen vectors were constructed via homologous recombination in yeast to obtain expression of fluorescent proteins in *Armillaria mellea* and to evaluate heterologous expression requirements in this basidiomycete (Fig. 1). All constructed vectors contained *hph* for transformant selection under the regulatory control of the *Agaricus bisporus gpdII* promoter and CaMV 35S terminator from pBGgHg, as this selection cassette had successfully generated *A. mellea* transformants previously^15^. Fluorescence cassettes were terminated by the *Aspergillus nidulans trpC* terminator. To determine efficacy of promoters and ascertain whether introns were required for expression in *A. mellea*, constructed vectors were identical except where the *P. chrysosporium* or *A. mellea gpd* promoters and 5′ introns were varied in fluorescence cassettes.

Functionality of constructed vectors was assessed by *Agrobacterium tumefaciens*–mediated transformation of *Clitopilus passeckerianus*, a readily-transformable Agaricomycete for which some requirements for transgene expression have been determined, and for which the *hph* selection cassette from pBGgHg is known to be functional^25^. GFP and mRFP expression was observed in hygromycin-resistant *C. passeckerianus* colonies that had been transformed with vectors containing 5′ introns, confirming functionality of the *P. chrysosporium* and *A. mellea gpd* promoters and *A. mellea* introns used in constructed vectors (Table 1). Vectors containing an atypical intron and a truncated intron (pCAM-hph-LiGFP and pCAM-hph-simRFP, respectively) did not confer GFP or mRFP expression in *C. passeckerianus.*

### Heterologous expression in *A. mellea*

*In vitro*-produced basidiospores of *A. mellea* ELDO17 were transformed using *A. tumefaciens* LBA1126 or AGL-1 carrying the intronless vector pBGgHg, two vectors containing 5′ introns that have conferred GFP expression in other basidiomycetes (pGR4-GFP where eGFP is driven by the *A. bisporus gpdII* promoter and pGR4-4iGM3 where eGFP is controlled by the *P. chrysosporium gpd* promoter; both have the *P. chrysosporium mnp* 3′ terminator) and each of the fourteen vectors constructed.

**Figure 2:**
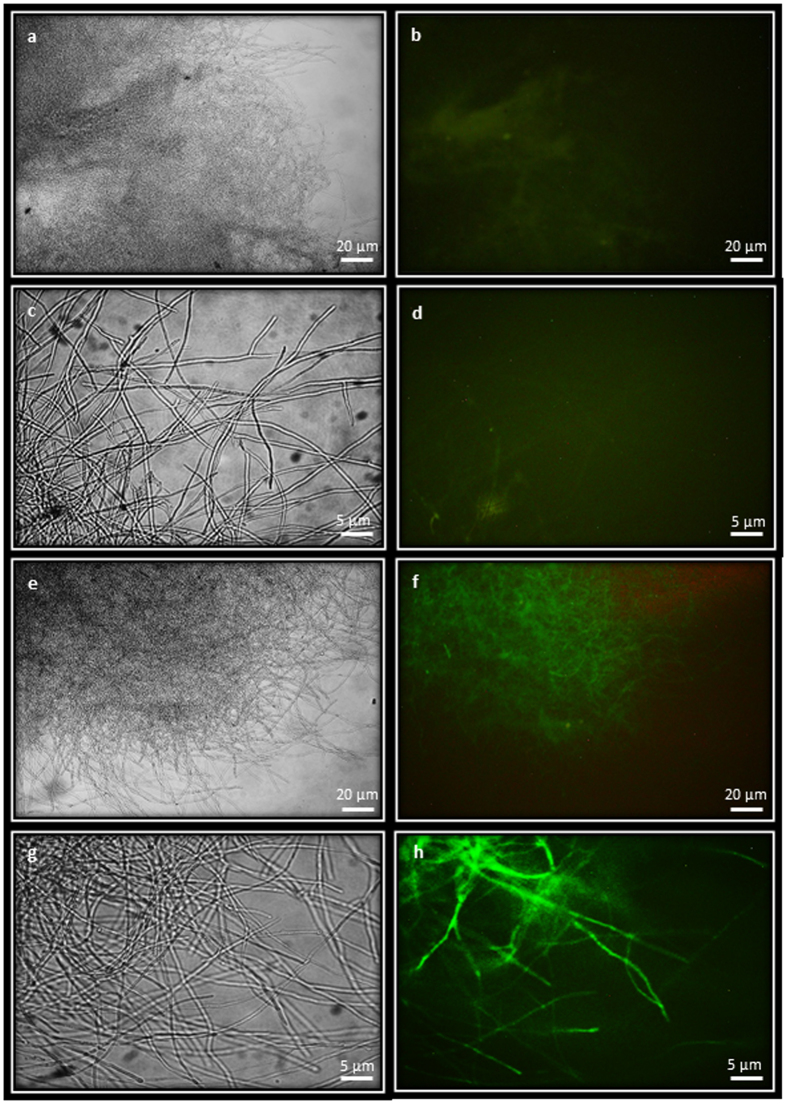
GFP expression in *Armillaria mellea.* *Armillaria mellea* ELDO17 WT mycelia viewed under bright field (**a**,**c**) and viewed with the GFP epifluorescent filter showing limited autofluorescence (**b**,**d**). *Armillaria mellea* transformed with pCAM-hph-Amgpd-xiGFP (transformant ELDO17-Amgpd-xiGFP2) viewed under bright field (**e**,**g**) and viewed with GFP epifluorescent filter showing bright GFP fluorescence (**f,h**). Mycelia were examined using a ×10 objective lens in (**a,b,e,f**) and with a ×40 objective lens in (**c,d,g,h**).

**Figure 3:**
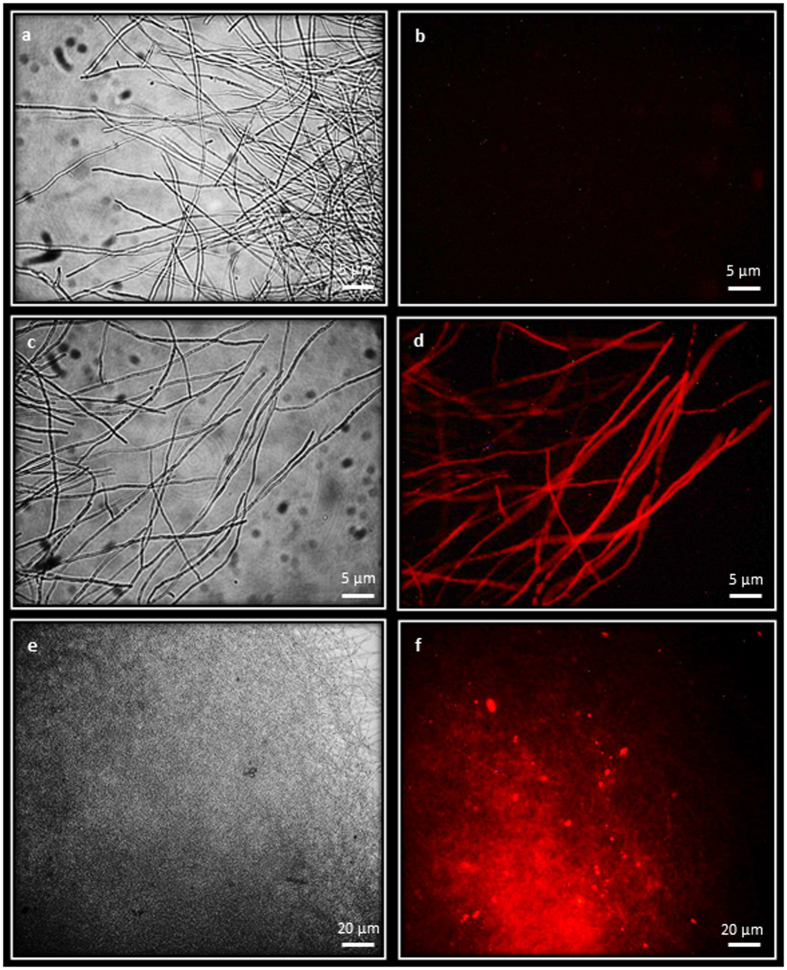
mRFP expression in *Armillaria mellea.* *Armillaria mellea* ELDO17 WT mycelia viewed under (**a**) bright field and (**b**) viewed with the mRFP epifluorescent filter showing no fluorescence. *Armillaria mellea* transformed with pCAM-hph-Amgpd-ximRFP (transformant ELDO17-Amgpd-ximRFP1) viewed under bright field (**c,e**) and viewed with mRFP epifluorescent filter showing bright, consistent mRFP fluorescence (**d,f**). Mycelia were examined using a x40 objective lens in (**a–d**) and with a ×10 objective lens in (**e,f**).

PCR analysis confirmed presence of *hph*, GFP and mRFP in hygromycin-resistant colonies transformed with the various vectors. All hygromycin-resistant colonies were analysed microscopically for fluorescence. Wild type ELDO17 mycelium, which only displayed weak, yellowish autofluorescence, was used as a comparison. Transformation using vectors where the reporter gene was regulated by the *A. mellea gpd* promoter and contained a 5′ intron from either *P. chrysosporium* or *A. mellea* (pCAM-hph-Amgpd-iGFP and pCAM-hph-Amgpd-xiGFP) gave strong and bright GFP expression in 25% of the transformants. Similarly, the equivalent mRFP plasmids (pCAM-hph-Amgpd-imRFP and pCAM-hph-Amgpd-ximRFP) conferred mRFP expression in 60% of transformants (Table 1). These four plasmids also conferred strong, bright fluorescent protein expression in *C. passeckerianus* (Table 1). In contrast, no fluorescence was readily detectable in colonies transformed with vectors where the *A. bisporus* or *P. chrysosporium gpd* promoters were driving mRFP or GFP, with or without 5′ introns. Even with the *A. mellea gpd* promoter controlling the fluorescence cassette, in vectors without a 5′ intron in the expression cassette there was no discernible fluorescence. This indicates the importance of both the endogenous *gpd* promoter and an intron for expression of fluorescent proteins in *A. mellea*. GFP expression observed in *A. mellea* colonies transformed with plasmid pCAM-hph-Amgpd-xiGFP is shown in Fig. 2 and mRFP expression with plasmid pCAM-hph-Amgpd-ximRFP is shown in Fig. 3.

The GFP and mRFP fluorescence was bright and stable in *A. mellea* mycelium through subsequent subculturing and fluorescence was visible in white, submerged rhizomorphs growing in liquid or agar PD medium. Fruiting was induced in two morphologically diploid transformants that strongly expressed GFP or mRFP in their mycelia (ELDO17-Amgpd-xiGFP2 and ELDO17-Amgpd-ximRFP1, respectively) and immature fruiting bodies were obtained in similar timescales to that described for the wild type^16^. Fluorescence was observed in primordia and in stipe, pileus and gill tissue of the immature fruiting bodies and in mycelial cultures derived from these tissues.

**Figure 4:**
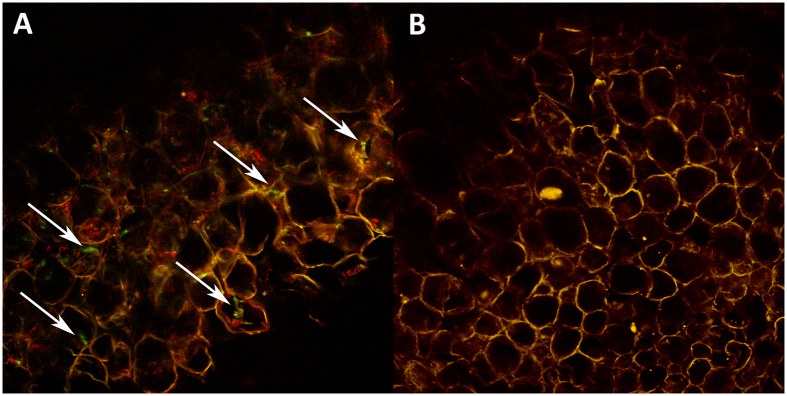
Confocal microscopy of root sections treated with antiGFP-AF 488. Visualised using a ×100 objective lens. Arrows show hyphae (green) of GFP-expressing isolate ELDO17-Amgpd-xiGFP2 (**A**). Hyphae of non-expressing isolate ELDO17-siGFP1 were not visible at the same wavelength, 488 nm (**B**). Although most plant cell walls were typically red, some were yellow (**A,B**), due to autofluorescence at 488 nm, which was visible in roots inoculated with both GFP, mRFP, and non-expressing isolates, regardless of treatment.

Confocal microscopy was used to visualise GFP and mRFP fluorescence *in planta* in walnut plants grown *in vitro.* Plants were inoculated with one GFP-expressing transformant (ELDO17-Amgpd-xiGFP2), one mRFP-expressing transformant (ELDO17-Amgpd-ximRFP1) and one non-GFP-expressing transformant (ELDO17-siGFP1). Non-inoculated plants served as controls. Among all inoculated plants, the medium was completely colonized by *A. mellea* by two weeks post-inoculation, based on the presence of the mycelium in the medium as viewed from the base of each Magenta box. At six weeks post-inoculation, approximately half of the plants per Magenta box succumbed to infection and the remaining living plants were symptomatic (Supplementary Fig. S1). The pathogen was recovered in culture from roots of all inoculated plants (with all three isolates), but not from non-inoculated plants, as expected (Supplementary Fig. S2). Under confocal fluorescence, hyphae of GFP-expressing and mRFP-expressing isolates were not visible in non-treated roots, but were visible in roots treated with WGA-AF 488, which binds to chitin in fungal cell walls. ELDO17-Amgpd-xiGFP2 was visible in root sections treated with antiGFP-AF 488, demonstrating GFP expression *in planta* (Fig. 4A). ELDO17-Amgpd-ximRFP1 was not visible in roots treated with antiGFP-AF 488, as expected. The non-expressing transformant ELDO17-siGFP1 was not visible in non-treated roots or in those treated with antiGFP-AF 488 (Fig. 4B), but was visible in roots treated with WGA-AF 488. Hyphae were not visible in non-treated or treated roots of non-inoculated plants.

## Discussion

There is relatively little information regarding the mechanism of gene expression in Agaricomycetes. Most of the information that is available has been inferred from model animal systems^51^,^52^,^53^ or from ascomycetes and basidiomycetous yeasts^54^,^55^,^56^. One of the most prominent prerequisites for efficient expression in some Agaricomycetes is the presence of an intron, reported in several such fungi including *Coprinopsis cinerea*^23^,^24^, *P. chrysosporium*^28^, *A. bisporus*^23^ and *Flammulina velutipes*^34^. An intron is not always essential for expression, however, and other studies have reported expression of fluorescent proteins using intronless constructs, for example in *Hebeloma cylindrosporum*^57^, *Ganoderma lucidum*^26^ and *Grifola frondosa*^58^. In this work, we have confirmed that *A. mellea* requires an intron for effective expression of GFP and mRFP, as no fluorescence was detected in colonies that were transformed with intronless vectors. Our fluorescent strains are stable, with fluorescence visible throughout fruiting and GFP expression detected during *in vitro* infection of walnut. The absence of an intron is likely to be one of the reasons GFP expression was not observed in transformants generated with the intronless pBGgHg in previous work^15^ and replicated here.

Whilst the requirement for introns for efficient expression in some Agaricomycetes is well-established^23^,^27^,^34^, precise mechanisms for intron splicing and transcript processing remain to be fully elucidated. Evaluation of model animal systems has shown that information required for correct intron splicing is usually contained within the intron itself and within adjacent exonic regions^52^,^53^, but differences between animal and fungal systems have been highlighted, for example, in a bioinformatic study that focused mainly on ascomycetes, Kupfer *et al*.^55^ demonstrated that the polypyrimidine tract for binding of spliceosomes that mediate intron excision is primarily between the 5′ donor splice site and the lariat intermediate branch site, rather than near the 3′ acceptor splice site as shown in animal systems. In general, the 5′ and 3′ splice sites and the branch site are highly conserved in fungi, but there is limited nucleotide conservation at other positions within the intron^55^ and experimentally, artificial introns constructed with random sequences outside of these conserved sites have conferred expression in *Schizophyllum commune*^27^. In this study, *C. passeckerianus* was transformed to assess vector functionality. *Clitopilus passeckerianus* has been previously shown to require introns for expression^25^ and this was confirmed here, with only intron-containing vectors conferring fluorescence. For two vectors however, the presence of an intron alone was insufficient for transgene expression. Plasmids pCAM-hph-simRFP and pCAM-hph-siGFP are identical except the former has a 2 bp deletion (TT) at positions 12 and 13 in the 52 bp intron. Only pCAM-hph-siGFP containing the intact intron conferred expression. The deletion in the pCAM-hph-simRFP intron is outside of the donor, acceptor and branch sites, but is potentially within the polypyrimidine tract and it is therefore possible that a TT deletion in this GT-rich region could prevent spliceosome binding and subsequent splicing, thereby prohibiting expression. Similarly, plasmid pCAM-hph-LiGFP containing a long (195 bp) intron at the 5′ end of GFP also did not confer expression in *C. passeckerianus.* As this is the 11^th^ intron from an *A. mellea* putative efflux transporter, non-expression in colonies transformed with this vector is possibly due to problematic integration of a 3′ intron into a 5′ position, resulting in the loss of contextual information required for correct transcript splicing. Additionally, as the median length of introns in basidiomycetes is between 50 and 100 bp^13^,^48^,^55^,^59^, a 195 bp intron is atypical and may not be recognised by *C. passeckerianus*. Non-recognition of heterologous introns is not necessarily unusual, and has been reported in *Aspergillus orzyae*, an ascomycete often used in heterologous expression studies^60^.

Another factor that has been shown to influence efficient gene expression in basidiomycetes is the use of an effective promoter, with previous studies demonstrating that promoters vary in efficiency^26^,^32^,^33^. Heterologous promoters from *P. chrysosporium* and *A. bisporus* are functional in *A. mellea*, driving expression of *hph* to generate hygromycin-resistant transformants^15^,^16^, yet they were unable to drive expression of the fluorescence cassettes in this work. There are several reports of non-functional heterologous promoters in Agaricomycetes (e.g. ref. 23), but this appears to be the first incidence where promoters are capable of driving expression of some genes but not others - perhaps only the few integration events where the heterologous promoters happened to be functional generated *A. mellea* transformants. As reported *A. mellea* transformation rates are low^15^,^16^, the use of an endogenous promoter to drive the selection cassette may increase transformation efficiency and should be considered for future transformation work with this Agaricomycete.

This work has demonstrated stable GFP and mRFP expression in *A. mellea* and highlighted the importance of both a native promoter and an intron for successful expression. The fluorescent strains generated here could be useful if they were to be deployed to help further visualise *A. mellea* infection processes *in vivo*, which would assist in understanding the specific root penetration and tissue colonisation mechanisms that are yet to be fully elucidated in *Armillaria*. Such studies are warranted because knowledge of the infection process can inform breeding, disease detection and disease management. The use of *C. passeckerianus* to test vector functionality in this work was informative, as only vectors that were functional in *C. passeckerianus* were functional in *A. mellea*. In addition, the non-expression of GFP and mRFP using two intron-containing vectors highlighted some of the many unknown aspects of efficient transgene expression, which may be important for future heterologous expression work in Agaricomycetes.

## Additional Information

**How to cite this article**: Ford, K. L. *et al*. A native promoter and inclusion of an intron is necessary for efficient expression of GFP or mRFP in *Armillaria mellea*. *Sci. Rep.* **6**, 29226; doi: 10.1038/srep29226 (2016).

## Supplementary Material

Supplementary Information

## Figures and Tables

**Table 1:** *Armillaria mellea* and *Clitopilus passeckerianus* transformants obtained with various vectors and the percentage showing expression of GFP or mRFP.

Vector	Promoter	Intron	Number of *C. passeckerianus* transformants (% expressing GFP/mRFP)	Number of *A. mellea* transformants (% expressing GFP/mRFP)
pBGgHg^30^	*Agaricus bisporus gpdII*	—	29 (0%)	31 (0%)
pGR4-4iGM3^23^	*A. bisporus gpdII*	*P. chrysosporium gpd*	n/a	27 (0%)
pGR4-GFP^23^	*Phanerochaete chrysosporium gpd*	*P. chrysosporium gpd*	n/a	23 (0%)
pCAM-hph-GFP	*P. chrysosporium gpd*	—	6 (0%)	10 (0%)
pCAM-hph-mRFP	*P. chrysosporium gpd*	—	11 (0%)	23 (0%)
pCAM-hph-Amgpd-GFP	*Armillaria mellea gpd*	—	5 (0%)	10 (0%)
pCAM-hph-Amgpd-mRFP	*A. mellea gpd*	—	13 (0%)	11 (0%)
pCAM-hph-LiGFP	*P. chrysosporium gpd*	*A. mellea et*^a^	6 (0%)	10 (0%)
pCAM-hph-siGFP	*P. chrysosporium gpd*	*A. mellea et*^b^	4 (100%)	17 (0%)
pCAM-hph-simRFP	*P. chrysosporium gpd*	*A. mellea et*^b^†	6 (0%)	11 (0%)
pCAM-hph-iGFP	*P. chrysosporium gpd*	*A. mellea gpd*	5 (40%)	10 (0%)
pCAM-hph-imRFP	*P. chrysosporium gpd*	*A. mellea gpd*	3 (33%)	16 (0%)
pCAM-hph-xiGFP	*P. chrysosporium gpd*	*P. chrysosporium gpd*	26 (96%)	18 (0%)
pCAM-hph-Amgpd-iGFP	*A. mellea gpd*	*A. mellea gpd*	4 (100%)	10 (20%)
pCAM-hph-Amgpd-imRFP	*A. mellea gpd*	*A. mellea gpd*	1 (100%)	4 (75%)
pCAM-hph-Amgpd-xiGFP	*A. mellea gpd*	*P. chrysosporium gpd*	5 (60%)	10 (30%)
pCAM-hph-Amgpd-ximRFP	*A. mellea gpd*	*P. chrysosporium gpd*	4 (75%)	6 (50%)

All vectors constructed contain the *hph* cassette from pBGgHg, where hygromycin phosphotransferase is under regulatory control of the *Agaricus bisporus gpdII* promoter and CaMV 35S terminator. Vectors contain either eGFP and mRFP (suffixed GM3/GFP and mRFP, accordingly) and are under regulatory control of either the *Phanerochaete chrysosporium gpd* promoter or *Armillaria mellea gpd* promoter (as indicated) with the *Aspergillus nidulans trpC* terminator.

^a^Contains intron 11 from a putative *A. mellea* efflux transporter (EF547153).

^b^Contains intron 7 from a putative *A. mellea* efflux transporter (EF547152).

^†^Vector pCAM-hph-simRFP has a 2 bp (TT) deletion in the intron sequence at positions 12 and 13.
