# Supplementary material for: A native promoter and inclusion of an intron is necessary for efficient expression of GFP or mRFP in Armillaria mellea
Source: Sci Rep. 2016 Jul 7;6:29226. doi: 10.1038/srep29226 (PMC4935854; doi:10.1038/srep29226)
Supplement: Supplementary Information [file srep29226-s1.pdf]

A native promoter and inclusion of an intron is necessary for efficient expression of GFP or mRFP in *Armillaria mellea*

Kathryn L. Ford [a], Kendra Baumgartner [b], Béatrice Henricot [c1], Andy M. Bailey [a], Gary D. Foster [a]*

[a] School of Biological Sciences, Life Sciences Building, University of Bristol, 24 Tyndall Avenue, Bristol, BS8 1TQ, United Kingdom.

[b] United States Department of Agriculture-Agricultural Research Service, 363 Hutchison Hall, University of California, One Shields Avenue, Davis, CA 95616, United States.

[c] The Royal Horticultural Society, Wisley, Woking, Surrey, GU23 6QB, United Kingdom.

[1] Current address: Forest Research, Northern Research Station, Roslin, Midlothian, EH25 9SY, United Kingdom.

* Corresponding author: Tel: +44 (0) 117 3941178. Fax: +44 (0) 117 331 7985. E-mail: Gary.Foster@bristol.ac.uk

Supplementary Table S1 - Construction details for plasmids

Plasmid	Primers	Fragments used to make plasmids and their sources
**pCAM-hph-GFP**	1F + 2R	*hph* cassette from pBGgHg
	3F + 4R	*Phanerochaete chrysosporium gpd* promoter from pGR4-GFP
	7BF + 8R	eGFP from pGR4-4iGM3
	9F + 10R	*Aspergillus nidulans trpC* terminator from pGR4-4iGM3
**pCAM-hph-mRFP**	1F + 2R	*hph* cassette from pBGgHg
	3F + 4R	*P. chrysosporium gpd* promoter from pGR4-GFP
	7AF + 8AR	mRFP from pYES-hph-RFP004
	9F + 10R	*A. nidulans trpC* terminator from pGR4-4iGM3
**pCAM-hph-Amgpd-GFP**	1F + 2R	*hph* cassette from pBGgHg
	A + B	1 kb *gpd* promoter from *Armillaria mellea* ELDO17 (protein ID 13125)
	C + 10R	eGFP through *A. nidulans trpC* terminator from pCAM-hph-siGFP
**pCAM-hph-Amgpd-mRFP**	1F + 2R	*hph* cassette from pBGgHg
	A + B	1 kb *gpd* promoter from *A. mellea* ELDO17 (protein ID 13125)
	M + 10R	mRFP through *A. nidulans trpC* terminator from pCAM-hph-simRFP
**pCAM-hph-LiGFP**	1F + 2R	*hph* cassette from pBGgHg
	3F + 4R	*P. chrysosporium gpd* promoter from pGR4-GFP
	5F + 6R	Intron from *A. mellea* ELDO17 (EF547153; intron 11)
	7F + 8R	eGFP from pGR4-4iGM3
	9F + 10R	*A. nidulans trpC* terminator from pGR4-4iGM3
**pCAM-hph-siGFP**	1F + 2R	*hph* cassette from pBGgHg
	3F + 4R	*P. chrysosporium gpd* promoter from pGR4-GFP
	5AF + 6AR	Intron from *Armillaria mellea* DSM3731 (EF547152; intron 7)
	7F + 8R	eGFP from pGR4-4iGM3

	9F + 10R	*A. nidulans trpC* terminator from pGR4-4iGM3
**pCAM-hph-simRFP**	1F + 2R	*hph* cassette from pBGgHg
	3F + 4R	*P. chrysosporium gpd* promoter from pGR4-GFP
	5AF + 6BR	Intron from *Armillaria mellea* DSM3731 (EF547152; intron 7)
	7CF + 8AR	mRFP from pYES-hph-RFP004
	9F + 10R	*A. nidulans trpC* terminator from pGR4-4iGM3
**pCAM-hph-iGFP**	1F + 4R	*hph* cassette through *P. chrysosporium gpd* promoter from pCAM-hph-siGFP
	K + L	Primer dimer of 1st intron from *A. mellea gpd* (protein ID 13125)
	E + 10R	eGFP through *A. nidulans trpC* terminator from plasmid pCAM-hph-siGFP
**pCAM-hph-xiGFP**	1F + 2R	*hph* cassette from pBGgHg
	3F + 8R	*P. chrysosporium gpd* promoter, intron/exon region & eGFP from pGR4-GFP
	9F + 10R	*A. nidulans trpC* terminator from pGR4-4iGM3
**pCAM-hph-imRFP**	1F + 4R	*hph* cassette through *P. chrysosporium gpd* promoter from pCAM-hph-siGFP
	K + Q	Primer dimer of 1st intron from *A. mellea gpd* (protein ID 13125)
	N + 10R	mRFP through *A. nidulans trpC* terminator from pCAM-hph-simRFP
**pCAM-hph-Amgpd-iGFP**	1F + 2R	*hph* cassette from pBGgHg
	A + D	1 kb *A. mellea* ELDO17 *gpd* promoter through to 1st intron (protein ID 13125)
	E + 10R	eGFP through *A. nidulans trpC* terminator from plasmid pCAM-hph-siGFP
**pCAM-hph-Amgpd-imRFP**	1F + 2R	*hph* cassette from pBGgHg
	A + D	1 kb *A. mellea* ELDO17 *gpd* promoter through to 1st intron (protein ID 13125)
	N + 10R	mRFP through *A. nidulans trpC* terminator from pCAM-hph-simRFP
**pCAM-hph-Amgpd-xiGFP**	1F + 2R	*hph* cassette from pBGgHg
	A + B	1 kb *gpd* promoter from *A. mellea* ELDO17 (protein ID 13125)
	R + 10R	Intron/exon region from *P. chrysosporium gpd* through eGFP and *A. nidulans trpC* terminator from pCAM-hph-xiGFP
**pCAM-hph-Amgpd-ximRFP**	1F + 2R	*hph* cassette from pBGgHg
	A + B	1 kb *gpd* promoter from *A. mellea* ELDO17 (protein ID 13125)
	R + S	Intron/exon region from *P. chrysosporium gpd* from pCAM-hph-xiGFP
	T + 10R	mRFP through *A. nidulans trpC* terminator from pCAM-hph-simRFP

pBGgHg was constructed by Chen *el al*. (2000)[30], pGR4-GFP and pGR4-4iGM3 were constructed by Burns *et al*. (2005)[23] and pYES-hph-RFP004 was constructed by Collins *et al*. (2010)[24].

Supplementary Table S2 – Details of primers used during vector construction

Primer	Direction	Sequence	Fragments used to make plasmids and their sources	Primer binding site
**IF**	F	TGGGCCCGGCGCGCCGAATTCCCGGGGATC ACTGGATTTTGGTTTTAGGAATTAGAAATT	*hph* cassette from pBGgHg	Left border / CaMV 35S terminator
**2R**	R	GAAGAAGAATTCAGAGGTCCGCAAGTAGAT	*hph* cassette from pBGgHg	*A. bisporus gpdII* promoter
**3F**	F	ATCTACTTGCGGACCTCTGAATTCTTCTTCG CATCTATTCGTGCCGAGAACCGGGCAAGC	*P. chrysosporium gpd* promoter from pGR4-GFP	*A. bisporus gpdII* promoter / *P. chrysosporium gpd* promoter
**4R**	R	CGGCATGTTCAAGTAGTGTAGGGGTGGAGG	*P. chrysosporium gpd* promoter from pGR4-GFP	*P. chrysosporium gpd* promoter
**5F**	F	CCTCCACCCCTACACTACTTGAACATGCCGG TGTGTTGGTGTTGTTACCGGCGCAAGGTC	Intron from *A. mellea* ELDO17 (EF547153; intron 11)	*P. chrysosporium gpd* promoter / *A. mellea* intron
**5AF**	F	CCTCCACCCCTACACTACTTGAACATGCCG GTACGTTTCCATTATCTATACTTTGTCGAT	Intron from *A. mellea* DSM3731 (EF547152; intron	*P. chrysosporium gpd* promoter / *A. mellea*

		GGGCTGAGTGCCCGTGGGCTAG	7)	intron
**6R**	R	CCCCGGTGAACAGCTCCTCGCCCTTGCTCA CCTGCATTGTTCTCAAAAAATTGGAAGTTT	Intron from *A. mellea* ELDO17 (EF547153; intron 11)	*A. mellea* intron / eGFP
**6AR**	R	CCCGGTGAACAGCTCCTCGCCCTTGCTCACC TAGCCCACGGGCACTCAGCCCATCGACAAA GTATAGATAATGGAAACGTAC	Intron from *A. mellea* DSM3731 (EF547152; intron 7)	*A. mellea* intron / eGFP
**6BR**	R	GAACTCCTTGATGACGTCCTCGGAGGAGGC CTAGCCCACGGGCACTCAGCCCATCGACAA AGTATAGATAATGGAAACGTAC	Intron from *A. mellea* DSM3731 (EF547152; intron 7)	*A. mellea* intron / mRFP
**7F**	F	TGAGCAAGGGCGAGGAGCTGTTCACCGGGG	eGFP from pGR4-4iGM3	eGFP
**7AF**	F	CCTCCACCCCTACACTACTTGAACATGCCG ATGGCCTCCTCCGAGGACGTCATCAAGGAG	mRFP from pYES-hph-RFP004	*P. chrysosporium gpd* promoter / mRFP
**7BF**	F	CCTCCACCCCTACACTACTTGAACATGCCGG TGAGCAAGGGCGAGGAGCTGTTCACCGGG	eGFP from pGR4-4iGM3	*P. chrysosporium gpd* promoter / eGFP
**7CF**	F	GCCTCCTCCGAGGACGTCATCAAGGAGTTC	mRFP from pYES-hph-RFP004	mRFP
**8R**	R	TTACTTGTACAGCTCGTCCATGCCGAGAGT	eGFP from pGR4-4iGM3	eGFP
**8AR**	R	GTTTGATGATTTCAGTAACGTTAAGTGGAT TTAGGCGCCGGTGGAGTGGCGGCCCTCGGC	mRFP from pYES-hph-RFP004	mRFP gene / *A. nidulans trpC* terminator
**9F**	F	ACTCTCGGCATGGACGAGCTGTACAAGTA AATCCACTTAACGTTACTGAAATCATCAAAC	*A. nidulans trpC* terminator from pGR4-4iGM3	eGFP / *A. nidulans trpC* terminator
**10R**	R	TCTTAAAGCTTGGCTGCAGGTCGACGGATC GCGGCCGCCAGTGTGATGGATATCTGCAGA	*A. nidulans trpC* terminator from pGR4-4iGM3	*A. nidulans trpC* terminator / right border
**A**	F	ATCTACTTGCGGACCTCTGAATTCTTCTTC AATTGGGTAGATGTCCTGTAAGTGCTCACG	1 kb *gpd* promoter from *A. mellea* ELDO17 (protein ID 13125)	*A. bisporus gpdII* promoter / *A. mellea gpd* promoter
**B**	R	CATGATGATTGCAGAAGTGTAAGACGATGA	1 kb *gpd* promoter from *A. mellea* ELDO17 (protein ID 13125)	*A. mellea gpd* promoter
**C**	F	TCATCGTCTTACACTTCTGCAATCATCATG GTGAGCAAGGGCGAGGAGCTGTTCACCGGG	eGFP through *A. nidulans trpC* terminator from plasmid pCAM-hph-siGFP	*A. mellea gpd* promoter / eGFP
**D**	R	CTACGAGAATACAATGAATGAGTACAGATG	1 kb *A. mellea* ELDO17 *gpd* promoter through to 1st intron (protein ID 13125)	*A. mellea gpd* promoter & 1st intron
**E**	F	CATCTGTACTCATTCATTGTATTCTCGTAG GTGAGCAAGGGCGAGGAGCTGTTCACCGGG	eGFP through *A. nidulans trpC* terminator from plasmid pCAM-hph-siGFP	*A. mellea gpd* promoter & 1st intron / eGFP
**K**	F	ATCCTCCCTCCACCCCTACACTACTTGAAC ATGGTAGCGTCTCGTCGTTGCTTACGTATC ATCTGTACTCATTCATTGTATTCTCGTAG	Primer dimer of 1st intron from *A. mellea gpd* (protein ID 13125)	*P. chrysosporium gpd* promoter / *A. mellea gpd* & 1st intron
**L**	R	CCCGGTGAACAGCTCCTCGCCCTTGCTCAC CTACGAGAATACAATGAATGAGTACAGA TGATACGTAAGCAACGACGAGACGCTACCAT	Primer dimer of 1st intron from *A. mellea gpd* (protein ID 13125)	*A. mellea gpd* start codon & 1st intron / eGFP
**M**	F	TCATCGTCTTACACTTCTGCAATCATCATG GCCTCCTCCGAGGACGTCATCAAGGAGTTC	mRFP through *A. nidulans trpC* terminator from pCAM-hph-simRFP	*A. mellea gpd* promoter / mRFP
**N**	F	CATCTGTACTCATTCATTGTATTCTCGTAG GCCTCCTCCGAGGACGTCATCAAGGAGTTC	mRFP through *A. nidulans trpC* terminator from pCAM-hph-simRFP	*A. mellea gpd* promoter & 1st intron / mRFP
**Q**	R	GAACTCCTTGATGACGTCCTCGGAGGAGGC CTACGAGAATACAATGAATGAGTACAGATG ATACGTAAGCAACGACGAGACGCTACCAT	Primer dimer of 1st intron from *A. mellea gpd* (protein ID 13125)	*A. mellea gpd* promoter & 1st intron / mRFP gene

**R**	F	CTCATCGTCTTACACTTCTGCAATCATCATG CCGGTCAGTACACCACACAGCCCGACCGC	Intron/exon region from *P. chrysosporium gpd* through eGFP & *A. nidulans trpC* terminator from pCAM-hph-xiGFP	*A. mellea gpd* promoter / *P. chrysosporium gpd* intron/exon region
**S**	R	TGCTTTGACCTGGAAAGCGAAGTCAGCACG	Intron/exon region from *P. chrysosporium gpd* from pCAM-hph-xiGFP	*P. chrysosporium gpd* intron/exon region
**T**	F	CGTGCTGACTTCGCTTTCCAGGTCAAAGCA GCCTCCTCCGAGGACGTCATCAAGGAGTTC	mRFP through *A. nidulans trpC* terminator from pCAM-hph-simRFP	*P. chrysosporium gpd* intron/exon region / mRFP

pBGgHg is from Chen *et al*. (2000)[30], pGR4-GFP and pGR4-4iGM3 were constructed by Burns *et al*. (2005)[23] and pYES-hph-RFP004 was constructed by Collins *et al*. (2010)[24].

Supplementary Table S3 – Primers used to evaluate constructed vectors and transformed fungi

Primer	Sequence (5' to 3')	Description	Reference
LB forward	GACTGATGGGCTGCCTGTATCGAG	Amplifies region between LB and RB of pCAM-hph-series when testing recombinant *E. coli* colonies	16
RB reverse	GTGGTTGGCATGCACATACAAATG		
hph forward	GCGTGGATATGTCCTGCGGG	Amplifies 600 bp of *hph* gene to ascertain transgene presence	25
hph reverse	CCATACAAGCCAACCACGGC		
GFP forward	ACGGCGACGTAAACGGCC	Amplifies 600 bp of GFP to ascertain transgene presence	25
GFP reverse	GTGATCGCGCTTCTCGTT		
mRFP forward	GCCTCCTCCGAGGACGTCATCAAGGAGTCC	Amplifies 674 bp of mRFP to ascertain transgene presence	This paper
mRFP reverse	TTAGGCGCCGGTGGAGTGGCGGCCCTCGGC		

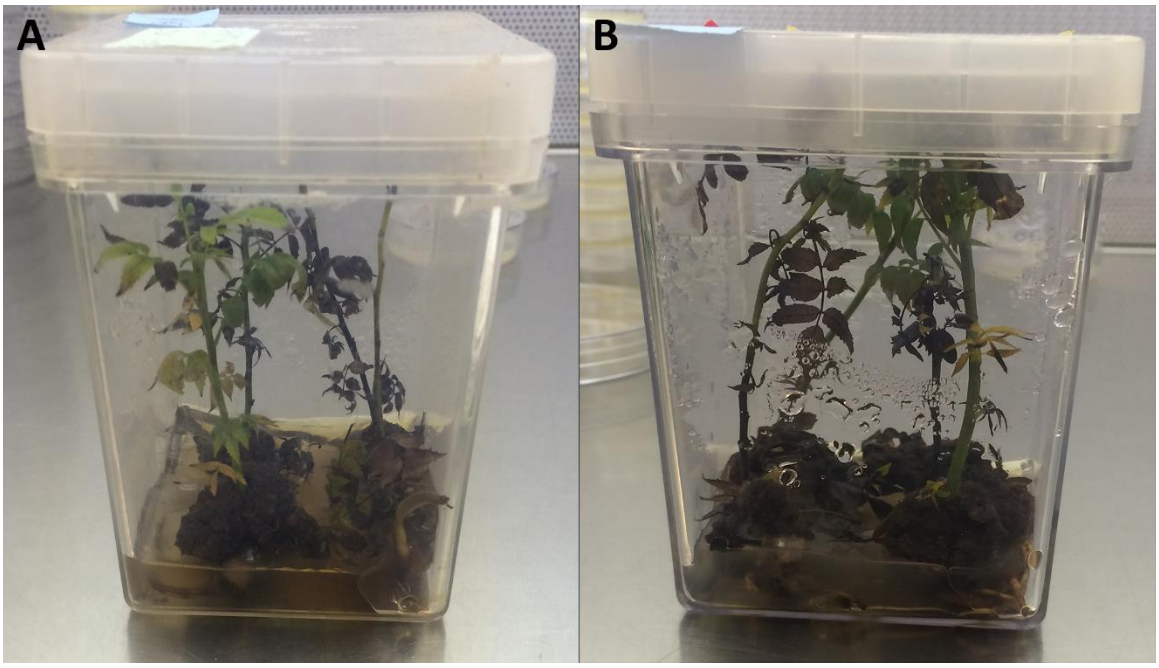

Supplementary Figure S1 – Plants inoculated with transformants ELDO17-Amgpd-xiGFP2 (A) and ELDO17-Amgpd-ximRFP1 (B), 6 weeks after inoculation. Between 4-6 weeks post-inoculation, leaves were either green with chlorotic/necrotic margins or completely brown and dead.

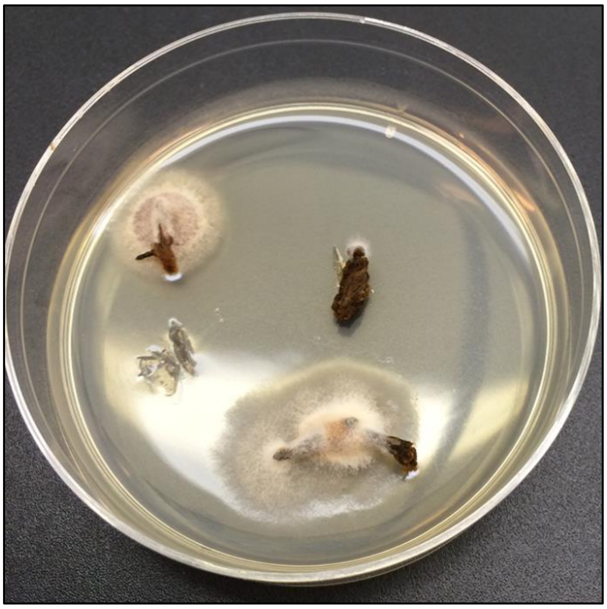

Supplementary Figure S2 – *Armillaria mellea* colonies recovered from three root fragments, which were sampled from a walnut plant inoculated with ELDO17-Amgpd-xiGFP2, 6 weeks after inoculation.
